# *In Vitro* Surfactant and Perfluorocarbon Aerosol Deposition in a Neonatal Physical Model of the Upper Conducting Airways

**DOI:** 10.1371/journal.pone.0106835

**Published:** 2014-09-11

**Authors:** Estibalitz Goikoetxea, Xabier Murgia, Pablo Serna-Grande, Adolf Valls-i-Soler, Carmen Rey-Santano, Alejandro Rivas, Raúl Antón, Francisco J. Basterretxea, Lorena Miñambres, Estíbaliz Méndez, Alberto Lopez-Arraiza, Juan Luis Larrabe-Barrena, Miguel Angel Gomez-Solaetxe

**Affiliations:** 1 Research Unit for Experimental Neonatal Respiratory Physiology, Cruces University Hospital, Barakaldo, Bizkaia, Spain; 2 Research Unit for Experimental Neonatal Respiratory Physiology, Cruces University Hospital, Barakaldo, Bizkaia, Spain; 3 Neonatal Intensive Care Unit, Cruces University Hospital, Barakaldo, Bizkaia, Spain; 4 Thermal and Fluids Engineering Division, Mechanical Engineering Department, TECNUN, University of Navarra, San Sebastian, Gipuzkoa, Spain; 5 Department of Physical Chemistry, Faculty of Science and Technology, University of the Basque Country, Leioa, Bizkaia, Spain; 6 Department of Electronics and Electrotechnics, High Technical School of Maritime Studies, University of the Basque Country, Bilbao, Bizkaia, Spain; University of Southern California, United States of America

## Abstract

**Objective:**

Aerosol delivery holds potential to release surfactant or perfluorocarbon (PFC) to the lungs of neonates with respiratory distress syndrome with minimal airway manipulation. Nevertheless, lung deposition in neonates tends to be very low due to extremely low lung volumes, narrow airways and high respiratory rates. In the present study, the feasibility of enhancing lung deposition by intracorporeal delivery of aerosols was investigated using a physical model of neonatal conducting airways.

**Methods:**

The main characteristics of the surfactant and PFC aerosols produced by a nebulization system, including the distal air pressure and air flow rate, liquid flow rate and mass median aerodynamic diameter (MMAD), were measured at different driving pressures (4–7 bar). Then, a three-dimensional model of the upper conducting airways of a neonate was manufactured by rapid prototyping and a deposition study was conducted.

**Results:**

The nebulization system produced relatively large amounts of aerosol ranging between 0.3±0.0 ml/min for surfactant at a driving pressure of 4 bar, and 2.0±0.1 ml/min for distilled water (H_2_Od) at 6 bar, with MMADs between 2.61±0.1 µm for PFD at 7 bar and 10.18±0.4 µm for FC-75 at 6 bar. The deposition study showed that for surfactant and H_2_Od aerosols, the highest percentage of the aerosolized mass (∼65%) was collected beyond the third generation of branching in the airway model. The use of this delivery system in combination with continuous positive airway pressure set at 5 cmH_2_O only increased total airway pressure by 1.59 cmH_2_O at the highest driving pressure (7 bar).

**Conclusion:**

This aerosol generating system has the potential to deliver relatively large amounts of surfactant and PFC beyond the third generation of branching in a neonatal airway model with minimal alteration of pre-set respiratory support.

## Introduction

Surfactant replacement therapy is a routine clinical practice for the prevention and treatment of infants with respiratory distress syndrome (RDS) [1]. Nevertheless, the need for tracheal intubation and mechanical ventilation, which are directly linked to lung injury and chronic lung disease [2], [3], represent a serious drawback at the time of initiating surfactant therapy. Therefore, the current trend in neonatology is towards the use of non-invasive respiratory support as a first line treatment for RDS [4], only indicating selective surfactant therapy in the cases that the continuous positive airway pressure (CPAP) is not effective enough. The beneficial effects of surfactant therapy have been shown to be maximized, however, if the therapy is applied early in the course of the disease [5], [6] and, hence for years, a method has been sought to administer surfactant during CPAP [7]–[9]. Likewise, tracheal intubation of patients is essential during partial liquid ventilation (PLV) [10] to proceed with perfluorocarbon (PFC) instillation, which together with the lack of success of PLV in the last acute RDS trial conducted in adults [11], represents a major disincentive to its clinical use in preterm infants.

Aerosol delivery in combination with CPAP holds potential to deliver surfactant or PFC with minimal manipulation of the airway and with less extensive hemodynamic changes due to the delivery being gradual [12], [13]. However, although the therapy has been considered to be safe, clinical studies to date on aerosolized surfactant remain inconclusive [7], [9], [14], [15]. Generally, therapeutic aerosols are generated extracorporeally and then aerosol particles have to be carried into the lungs by the inspiratory airflow, which favors upper airway deposition and significantly reduces the amount of aerosol reaching the distal lung. In preterm infants, these difficulties are compounded by their extremely low lung volumes, narrow airways (especially in disease states) and high respiratory rates [16]. In this regard, lung deposition fractions of less than 1% of the nominal dose have been reported during aerosol delivery to preterm neonates [17], [18]. Furthermore, the biophysical characteristics of the liquid compounds significantly affect the performance of the aerosol devices [19]. For instance, clinical surfactant preparations, which contain lipids and proteins, have a high viscosity and, therefore, long aerosolization pulses are required.

Intracorporeal aerosol delivery of surfactant and PFCs, on the other hand, has been shown to improve lung function to the same extent, or even further, than their tracheal instillation, likely due to increased lung deposition [20], [21]. These experimental studies, however, were performed in intubated animals, with the particle generating device, an intracorporeal inhalation catheter (IC), inserted into the endotracheal tube and its aerosol generating tip placed at the distal end, at the carina.

We hypothesize that with this type of system, an intracorporeal, yet non-invasive, surfactant or PFC aerosol could be generated in combination with nasopharyngeal CPAP, for instance. To explore this, we designed the present *in vitro* feasibility study in which we investigated: 1) variations in the aerosol production rate of surfactant and PFCs and their particle size distribution at different driving pressures (4–6 bar), 2) the lung deposition of surfactant and PFC aerosols in a polymer-based 3D model of the upper conducting airways of an infant, and 3) the air flow rate produced by the IC and the pressure the system contributed to pre-set CPAP, with a view to its potential clinical application in combination with non-invasive respiratory support.

## Materials and Methods

### Compounds

Poractant-alfa (Curosurf, Chiesi Farmaceutici S. P. A., Parma, Italy; density 1 g/ml; kinematic viscosity  = 10.5 cSt; surface tension between 0 and 30 dyne/cm) is a natural surfactant, prepared from porcine lungs, containing almost exclusively polar lipids, in particular phosphatidylcholine, and about 1% of specific low molecular weight hydrophobic proteins SP-B and SP-C at a phospholipid concentration of 80 mg/ml. In addition to surfactant, we used two different types of PFC: perfluorodecalin (PFD; C_10_F_18_, F2 Chemicals Ltd., Lancashire, UK; density at 25°C = 1.95 g/ml; kinematic viscosity  = 2.7 cSt; vapor pressure at 37°C = 14 mmHg; surface tension  = 15 dyne/cm) and FC-75 (C_8_F_16_O, Fluorinert, 3 M, Neuss, Germany; density at 25°C = 1.78 g/ml; kinematic viscosity  = 0.81 cSt; vapor pressure at 37°C = 63 mmHg; surface tension  = 15 dyne/cm). These PFC species have been previously tested in several experimental studies [22], [23]. Also, distilled water (H_2_Od, B. Braun, Melsungen, Germany; density  = 1 g/ml; kinematic viscosity  = 1.003 cSt; surface tension  = 73 dyne/cm) was used to simulate the aerosolization of aqueous medications.

### Aerosol delivery system

A pneumatic catheter control system (LABneb, Trudell Medical International, London, ON, Canada) operated at variable driving pressures (4–7 bar) and connected to an IC (AeroProbe, Trudell Medical International) was used to produce surfactant and PFC aerosols. In total, three ICs of the same type were used during the study; these catheters are 100 cm long and consist of six outer lumens with a proximal diameter of 330 µm and one inner lumen with the same proximal diameter. Detailed information of the design and operational principles of this type of system can be found elsewhere [24]. If this system is operated with saline, a liquid flow rate of approximately 2.5 ml/min is achieved with a mass median aerodynamic diameter (MMAD) of about 15 µm (data from the manufacturer).

### Distal air flow rate and pressure measurements during CPAP

The distal air flow rate (Q_air_) and the pressure added by three identical ICs were measured using a differential pressure transducer (range: 0–71 cmH_2_O, Honeywell, NJ) fitted to a pneumotachograph (Fleisch 000, Lausanne, Switzerland) with a measurement uncertainty of ±0.5%. While already connected to the catheter control system, the IC was inserted into an endotracheal tube with an internal diameter of 3 mm (Mallinckrodt Medical, St. Louis, MO) until the distal tip of the IC extended 1 mm beyond the end of the tube. A further tube, 100 mm in length and 12 mm in internal diameter, was placed between the distal end of the endotracheal tube and the entrance of the pneumotachograph to achieve a fully developed flow at the measurement point.

**Figure 1:**
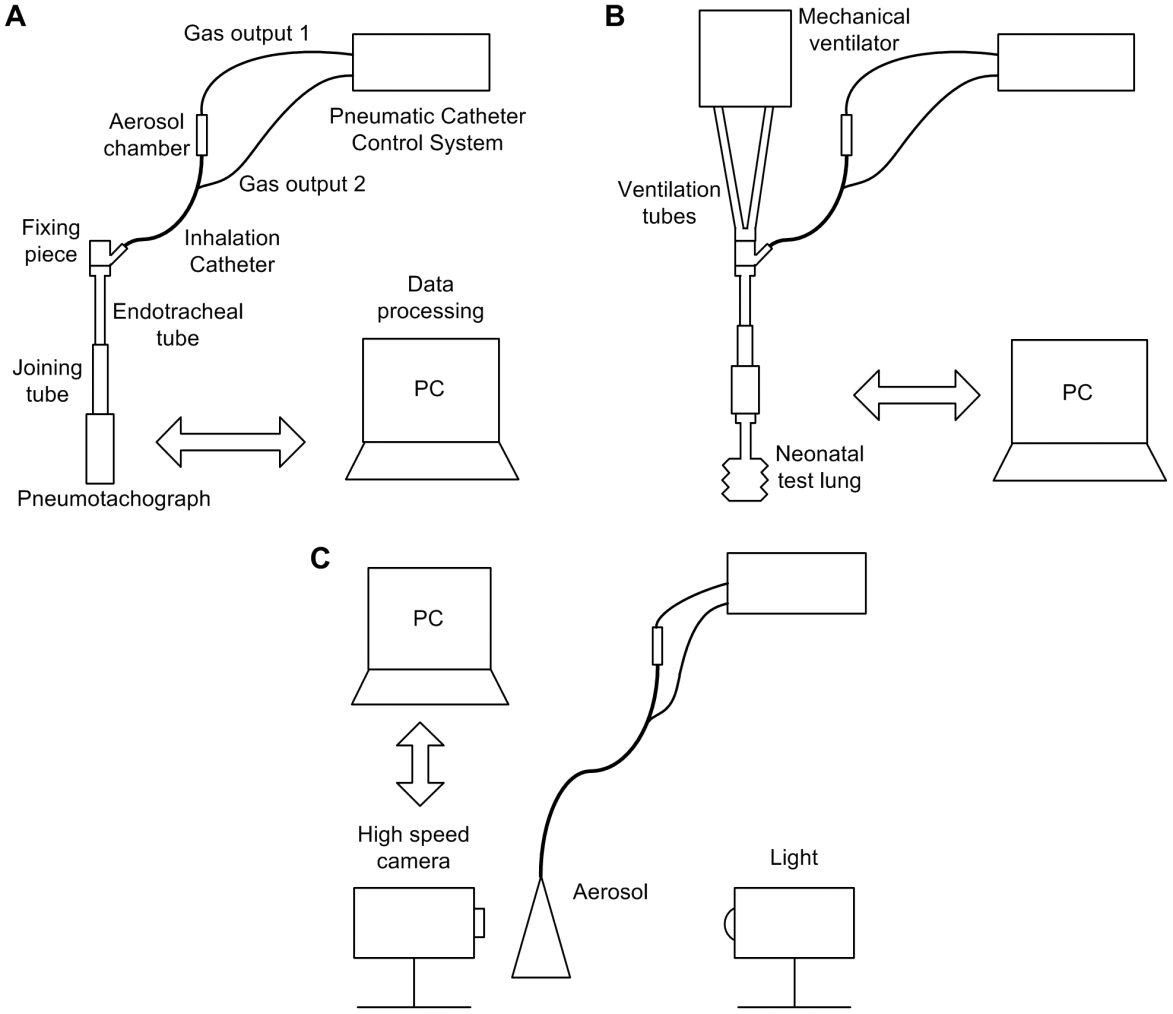
Experimental set-ups. A, Pneumotachograph equipment for air flow rate measurements. B, Measurement set-up for distal pressure. C, High speed camera equipment for studying the aerosol formation.

During Q_air_ measurements, the outlet of the pneumotachograph was left open and the catheter control system was triggered for 15 seconds. On the other hand, for the pressure measurements, the pneumotachograph was coupled to a synthetic neonatal test lung (Dräger Medical, Lübeck, Germany) and a CPAP of 5 cmH_2_O was generated by a neonatal mechanical ventilator (BP200, Beard Med Sys., Riverside, CA) at the proximal part of the endotracheal tube; then, the catheter control system was triggered for 15 seconds (Figure 1A, Figure 1B). This allowed us to study the maximum pressure achieved using the CPAP and the IC simultaneously and to determine the feasibility of using them together. The air flow generated by the IC and the pressure added to the CPAP were measured with the catheter control system operated at different driving pressures (4–7 bar). All the procedures were repeated at least five times. The data were recorded electronically (PowerLab/16SP and Chart 5 software, ADInstruments, Colorado Springs, CO).

### High speed camera analysis of surfactant and PFC aerosols

A high speed camera with associated software (MotionXtra HG-LE and MotionCentral software; Redlake, Tucson, AZ) were used to capture and record images of surfactant and PFC aerosols at 10,000 frames per second for 1.3 s. A long distance microscope (K2/SC Close-Focus Objective CF-1, Edmund Optics, Barrington, NJ) was attached to the high speed camera to obtain enlarged images near the tip of the IC. A continuous light source (Dedocool, Innovision Optics, Santa Monica, CA) illuminated the aerosol area to achieve images with a higher contrast between the aerosol and the background. Pixel dimensions of 144×304 (yielding a resolution of 6.5 µm/pixel) and a distance of 300 mm between the IC tip and the lens were set to ensure high quality recording (Figure 1C). A priori, images were recorded placing a ruler in parallel with the tip of the IC, to allow calculation of the length represented by each pixel in the digital images.

**Figure 2:**
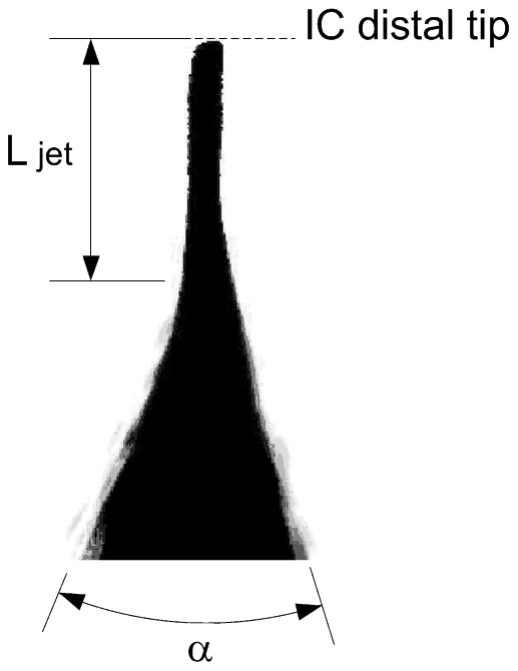
Image resulting from the superposition of 10,000 frames. α: aerosol angle. L_jet_: distance at which the aerosol cone is formed. IC: inhalation catheter.

MATLAB software (MathWorks, Natick, MA) was used for image processing. The mean diameter of the liquid jet before break-up (D_jet_) was measured selecting 20 frames from a total set of 13,000 frames captured for each compound and driving pressure (4–6 bar). This proportion was found to be sufficient to achieve results that were independent of the number of frames that had been selected. On the other hand, 10,000 frames (Figure 2) were superimposed to determine the aerosol cone angle (α) and the distance at which the aerosol cone was formed (L_jet_) for each compound and driving pressure. These parameters and the diameter of model trachea (D_trachea_) allowed the calculation of the collision length (L_col_), which is the distance that particles would travel before colliding against the trachea of the 3D model of the upper conducting airways, as follows:
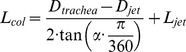



### Development of the infant airway physical model

**Figure 3:**
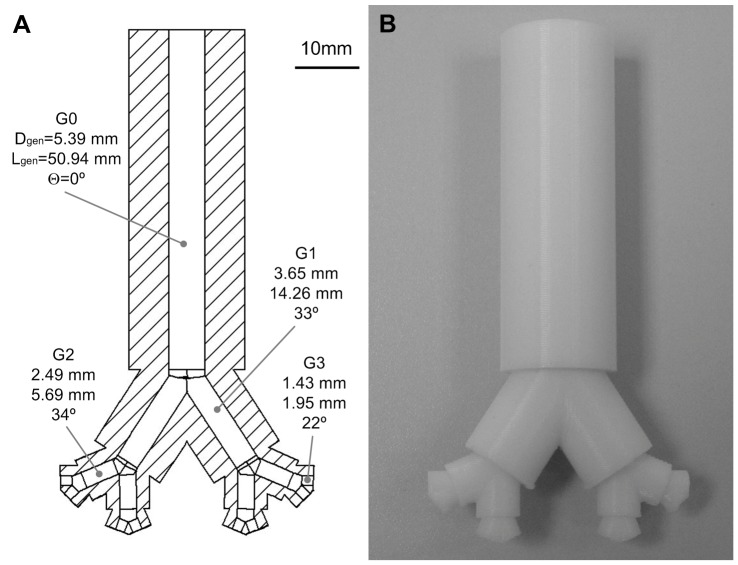
Infant upper conducting airway physical model. A, Drawing of the model created using computer-aided design (CAD) software. B, Photograph of the printed model. G0: trachea, G1: First generation, G2: Second generation, G3: Third generation, D_gen_: diameter of each generation, L_gen_: length of each generation, θ: branching angle between the airways of each generation.

A 3D printed model of the trachea and the first three generations of the airways of an infant was produced by rapid prototyping (Figure 3). The dimensions of the airway model were based on the morphometric study of a neonate described by Rozanek and Roubik [25]. The length of the trachea was considered 15 mm longer to allow the coupling between the IC tip and printed model for the measurements. Branching angles were obtained from a lung deposition study conducted by Yeh et al. [26]. With these data, an infant airway model was drawn using computer-aided design software (Solid Edge, Siemens PLM Software, Munich, Germany). Several 3D copies of the infant airway model were produced in Ivory ABS material (Stratasys, Eden Prairie, MN) with a 3D printer (HP DesignJet Color 3D printer, Hewlett-Packard Development Company, Houston, TX).

### Aerosol output and in vitro deposition in the infant airway model

**Figure 4:**
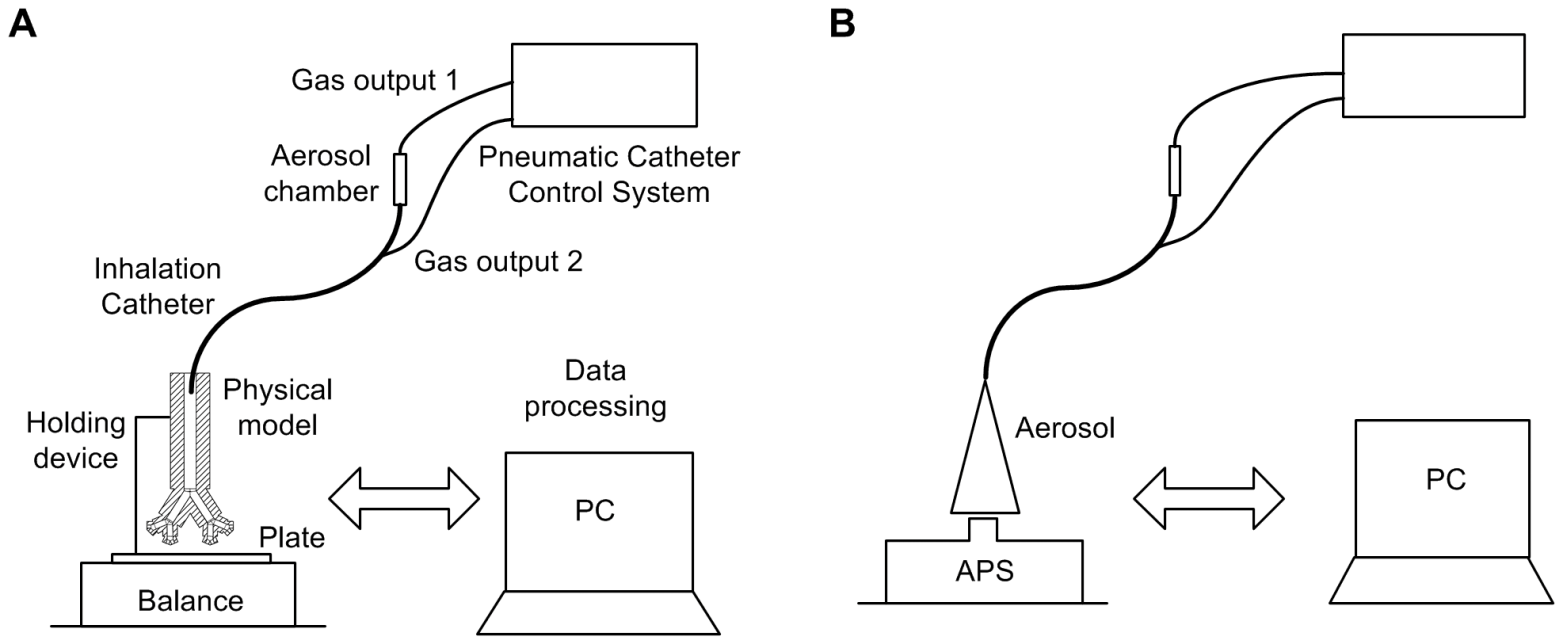
Experimental set-ups for the deposition study and particle size characterization. A, Equipment for aerosol production rate (AR) and *in vitro* deposition measurements (E_PM_, E_+3_, E_FP_). B, Aerodynamic Particle Sizer for particle size characterization.

Measurements were taken to assess the aerosol production rate of the IC for each compound at different driving pressures (range: 4–6 bar) and the deposition within and beyond the 3D model of the upper conducting airways. After connection of the IC to the catheter control system, its distal end was inserted into a rigid tube to allow vertical alignment with the proximal part of the 3D model (Figure 4A). Then, the rigid tube, containing the tip of the catheter, was inserted 1.5 cm into the printed model, simulating its placement just above the vocal cords. The 3-D infant airway model was mounted on a specially built support, holding it exactly 2 cm above a plate that was placed on a precision balance (AB54 Mettler-Toledo, Greifensee, Switzerland).

Each component of the set-up (compound-filled aerosol chamber, dry infant airway model and flat plate) was weighed before any aerosol generation. Once the pre-aerosolization weights were recorded, aerosolization was maintained for 40 s. Thereafter, each component was weighed again. The aerosolization rate (AR, ml/min) was calculated as the difference in weight of the aerosol chamber between before and after aerosolization divided by the pulse duration. The weight gain of the infant airway model and the flat plate were used to determine the mass flow rate for the compound emitted that was deposited in the airway physical model (E_PM_) and that advanced beyond the third generation branching (E_+3_), respectively. The difference between AR and the sum of E_PM_ and E_+3_ was attributed to the finest particles emitted by the IC (E_FP_). The procedure was repeated at least six times with each compound and each driving pressure (4–6 bar).

### Particle size characterization

The droplet size measurements in each case were made using an Aerodynamic Particle Sizer Spectrometer (APS 3321, TSI, Brooklyn, NY) with a resolution between 0.02 µm and 0.03 µm, for 1 µm and 10 µm diameter particles, respectively. Briefly, the aerosol particles are accelerated by suction through a nozzle on the top of the spectrometer; the size of each particle is calculated using the time taken to pass through two laser beams at a known fixed distance, i.e., the time-of-flight method. Due to higher inertia, particles with larger diameters travel at lower velocities. The device is designed to measure droplets with aerodynamic diameters between 0.5–20 µm.

To perform the measurements, the distal end of the IC was vertically aligned with the suctioning nozzle of the APS (Figure 4B). Thereafter, the optimal distance between the distal tip of the IC and the suctioning nozzle of the APS was determined for each compound, ensuring that the number of particles suctioned by the nozzle was within the range necessary to achieve an accurate measurement (1,000 particles/cm^3^). This optimal distance ranged between 5.2 and 50 cm depending on the compound being aerosolized and the driving pressure (4–7 bar). Thereafter, aerosolization with each compound and each driving pressure was maintained for 5 seconds. The aerosol particle size distributions were well approximated as log-normal distributions [27]. The mean aerodynamic diameter (D_a_), the MMAD and the geometric standard deviation (GSD) were analyzed.

### Statistical analysis

The mean (E_FP_, E_+3_, E_PM_, AR, MMAD, GSD and D_a_) ± standard deviation (SD) of the measurements were calculated for each compound and driving pressure. A one-way ANOVA (JMP8 software Statistical Discovery, SAS, NC) was performed to identify any statistically significant differences between E_FP_, E_+3_, E_PM_, and AR, with a p value of less than 0.05 being considered significant.

## Results

### Distal air flow rate and pressure measurements during CPAP

**Figure 5:**
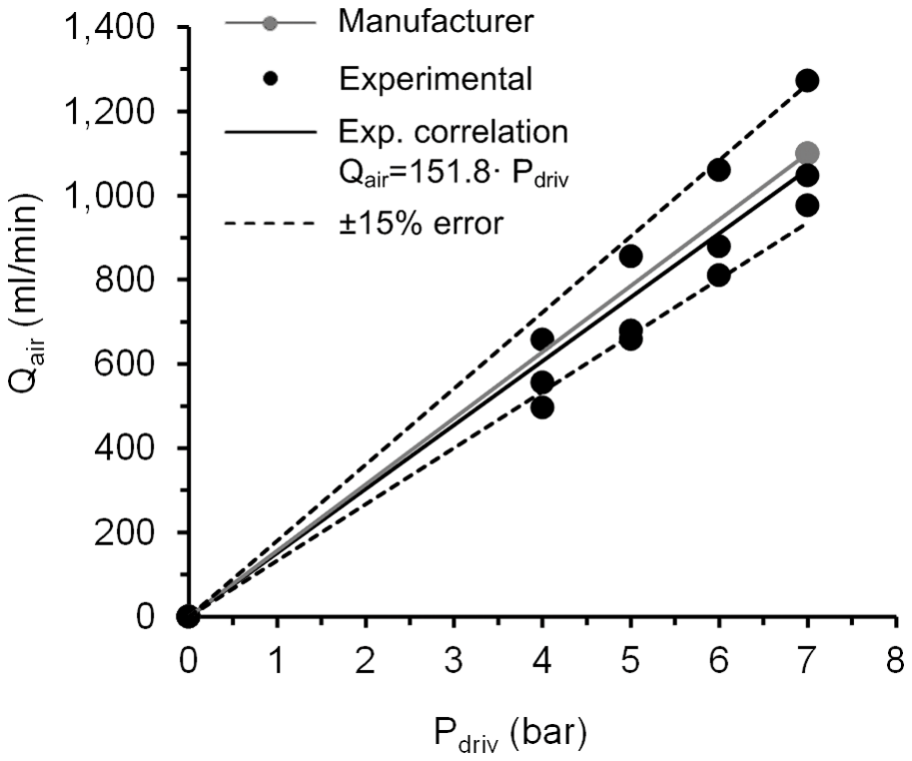
Air flow rate as a function of the driving pressure for three ICs. Comparison between experimental values of air flow rate that fit with a linear correlation and the values of the manufacturer; ±15% error obtained with respect to the data from the manufacturer. Q_air_: air flow rate. P_driv_: driving pressure.

A slight variability was found in terms of air flow rate with the use of the ICs (Figure 5). The air flow rate produced by the IC ranged between 496.1±0.5 ml/min (4 bar) and 1,272.7±1.1 ml/min (7 bar). Most of the values lay within ±15% of those provided by the manufacturer. A linear correlation was obtained between the driving pressure of the catheter control system and the air flow rate at the distal end of the ICs.

**Figure 6:**
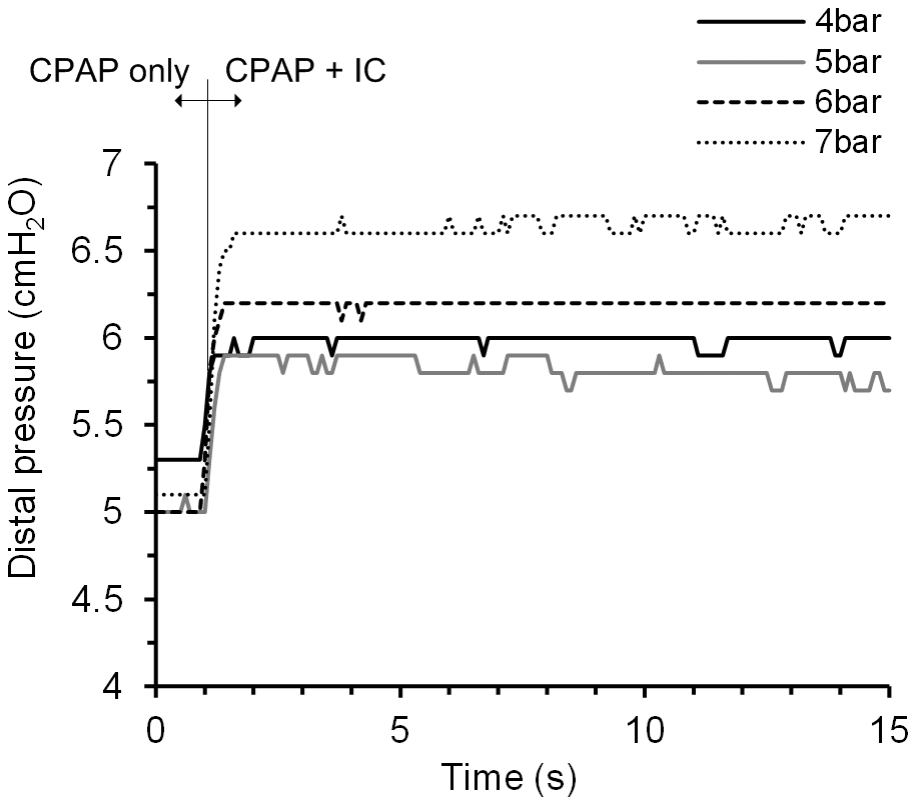
Air distal pressure as a function of the time and driving pressure for one IC. Pressure increase using the CPAP flow and a pulse of the IC simultaneously.CPAP: continuous positive airway pressure; IC: inhalation catheter.

Electronic recording during CPAP-only flow registered continuous pressure values between 5.0 and 5.5 cmH_2_O. The use of the ICs during this pre-set CPAP increased the recorded distal pressure as a function of the driving pressure used to power the catheter control system. The minimum pressure increases of 5.22 to 5.77±0.04 cmH_2_O were obtained with the catheter control system driven at 4 bar, and the maximum increases of 5.13 to 6.73±0.04 cmH_2_O with the highest driving pressure of 7 bar (Figure 6).

### High speed camera analysis of surfactant and PFC aerosols

**Figure 7:**
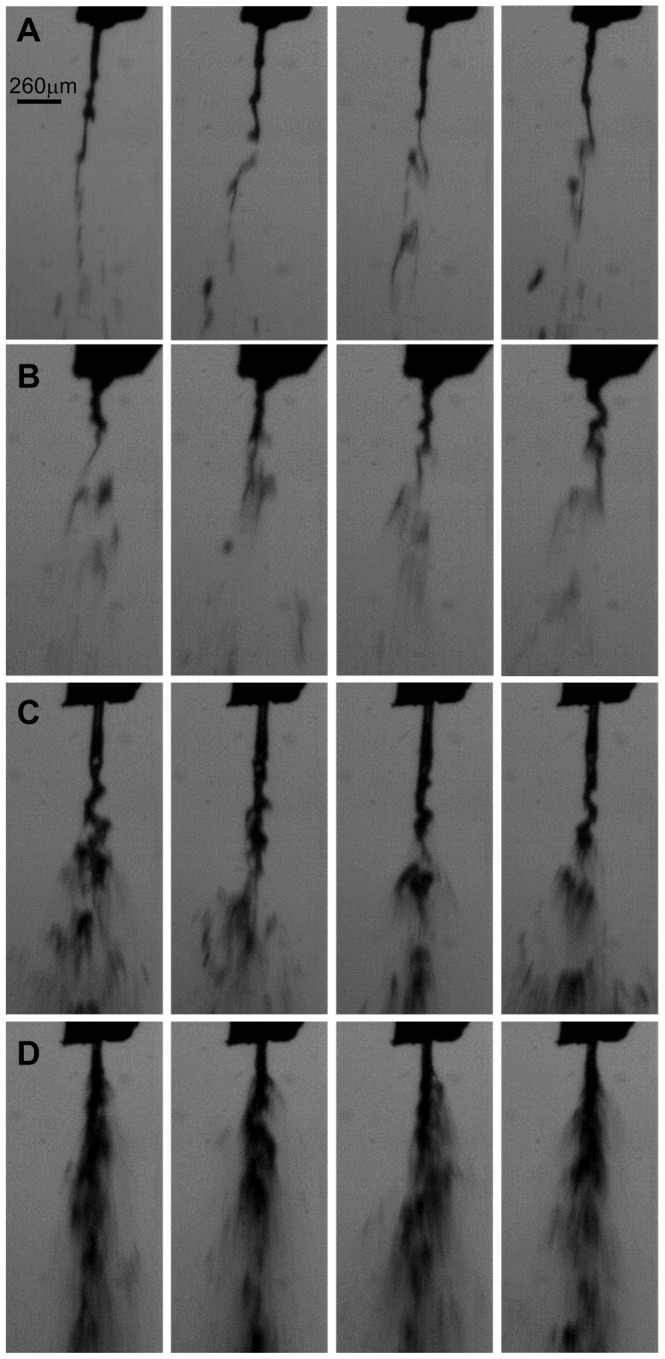
Representative images of aerosols at intervals of 0.1 ms. A, Surfactant at 4 bar. B, Surfactant at 6 bar. C, FC-75 at 4 bar. D, FC-75 at 6 bar.

**Figure 8:**
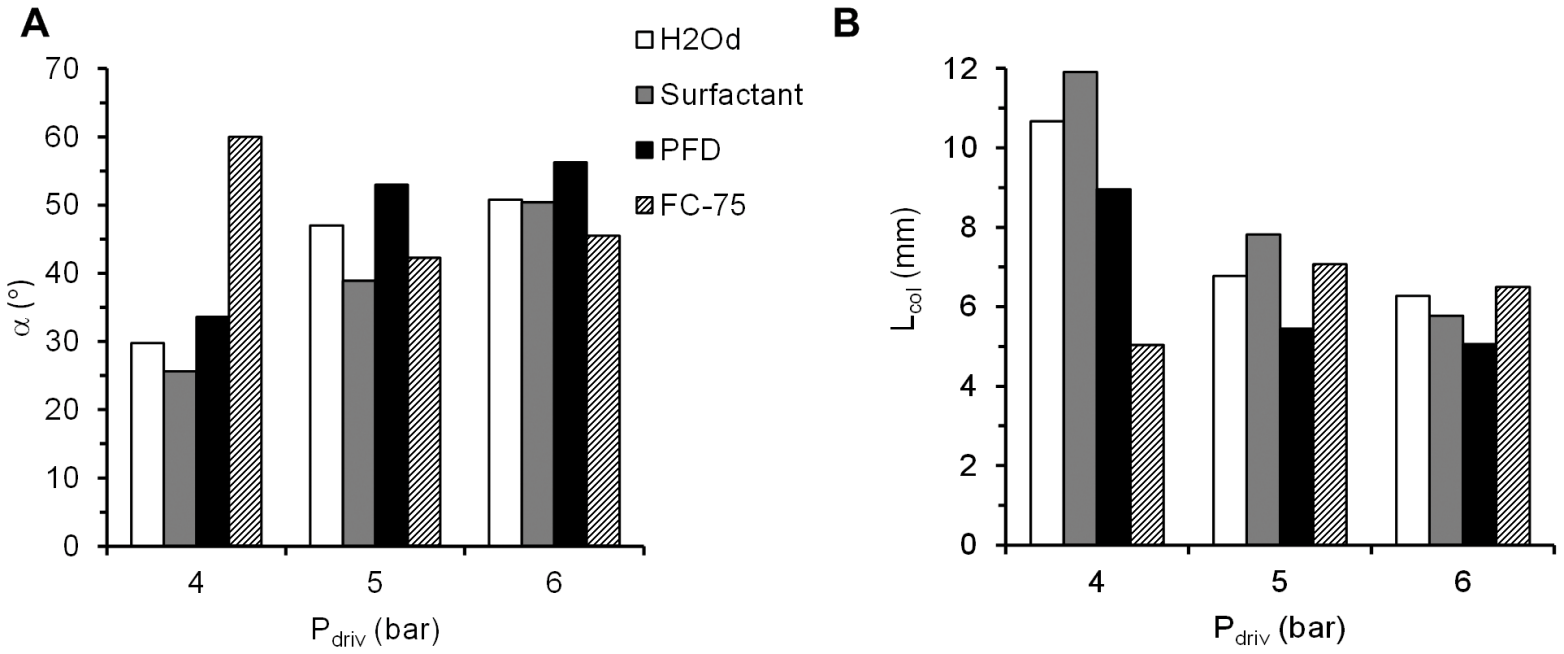
Variation of the main parameters in order to predict the impact between the particles and the physical model of the trachea, as a function of the aerosolized compound and the driving pressure. A, Collision length, L_col_. B, Aerosol cone angle, α. H_2_Od: distilled water. PFD: perfluorodecalin. P_driv_: driving pressure.

The phenomenon of aerosol generation was captured for every compound and driving pressure (Figure 7). An initial deformation of the liquid jet and its posterior break-up into droplets was successfully visualized with this method. In addition, the behavior of the aerosol within the trachea of the 3D model of the upper conducting airways of a neonate was estimated in terms of the collision length (L_col_), the distance which the particles of the aerosol cloud were predicted to travel before colliding with the model trachea, and the angle of the aerosol cone (α). L_col_ was the longest for H_2_Od and surfactant at low driving pressures of 4 bar (11 and 12 mm respectively) (Figure 8A), and shortest during PFC aerosolization, for PFD at 5 and 6 bar and for FC-75 at 4 bar (5 mm in these cases). The values of α were between 25.6° for surfactant and 59.9° for FC-75, at 4 bar in both cases (Figure 8B). Except in the case of FC-75, which is a highly volatile compound, a trend towards shorter L_col_ and larger α was observed after increasing the driving pressure. Regardless of the aerosolized compound, these results predict the formation of a surfactant or PFC film at the beginning of the trachea, if the IC is placed just above the vocal cords.

### Development of the infant airway physical model

In view of the small internal section of the distal generations of the 3D airway model, all printed models were tested with compressed air and water, in order to ensure the quality of the pieces and avoid potential orifice obstruction. Following this testing, fifteen valid copies of the model were selected to perform the surfactant and PFC deposition study.

### Aerosol output and *in vitro* deposition in the infant airway model

**Figure 9:**
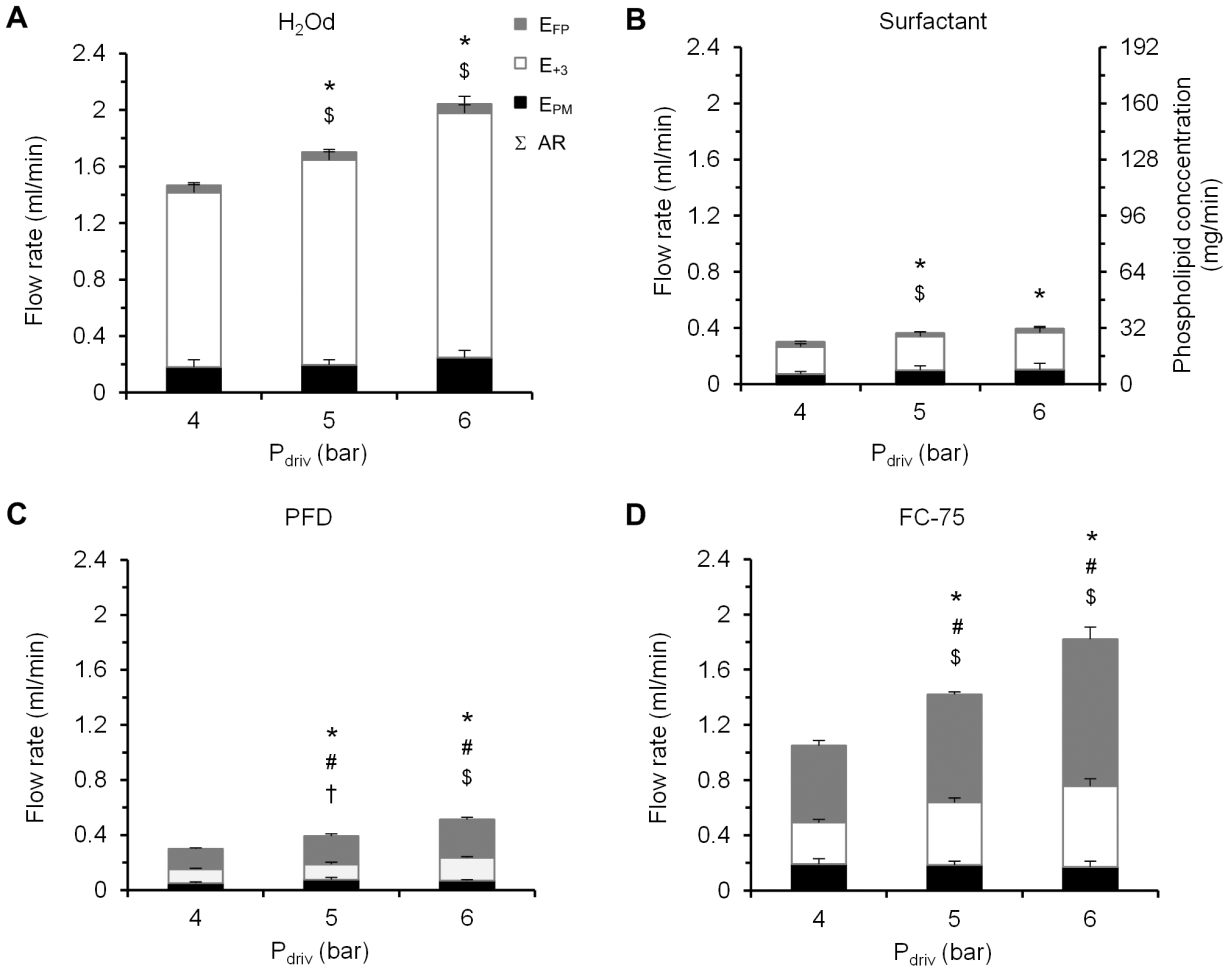
Deposition results as function of the compound and the driving pressure. A, Deposition results for H_2_Od; B, Surfactant; C, Perfluorodecalin, PFD; D, FC-75. Right vertical axis values on B are the phospholipid quantity (mg) corresponding to each rate on the left axis. E_FP_: mass flow rate of fine particles. E_+3_: mass flow rate that exits from the model. E_PM_: mass flow rate for the compound deposited within the model. AR: aerosolization rate. Σ: total height of each column. P_driv_: driving pressure. Values are given as mean ± SD. * is for AR vs. previous pressure; # is for E_FP_ vs. previous pressure; $ is for E_+3_ vs. previous pressure; and † is for E_PM_ vs. previous pressure. *p<0.05*, One-factor ANOVA.

The AR values ranged between 0.3±0.0 ml/min for surfactant at 4 bar and 2.0±0.1 ml/min for H_2_Od at 6 bar (Figure 9). Among the PFCs, the highest AR values corresponded to FC-75, irrespective of the driving pressure used to power the catheter control system. In the case of surfactant and H_2_Od, most of the output was collected beyond the third generation of branching as shown by the E_+3_ data, from 65.0±7.4% at 4 bar for surfactant to 85.2±3.4% at 5 bar for H_2_Od, whereas 23.7±6.4% and 11.5±2.1% of the output respectively was trapped within the model (E_PM_) and just 11.3±2.0% and 3.3±1.1% respectively was attributed to the fine particle of the aerosol (E_FP_). With regard to the PFC species, a significant amount of the aerosol was also collected beyond the third generation branching, although in this case, a higher percentage was attributed to fine particles (from 49.8±2.0% of the aerosolized mass at 4 bar for PFD to 58.5±4.9% at 6 bar for FC-75).

Regardless of the aerosolized compound, more mass was deposited on the plate (E_+3_) than within the model (E_PM_).

### Particle size characterization

**Figure 10:**
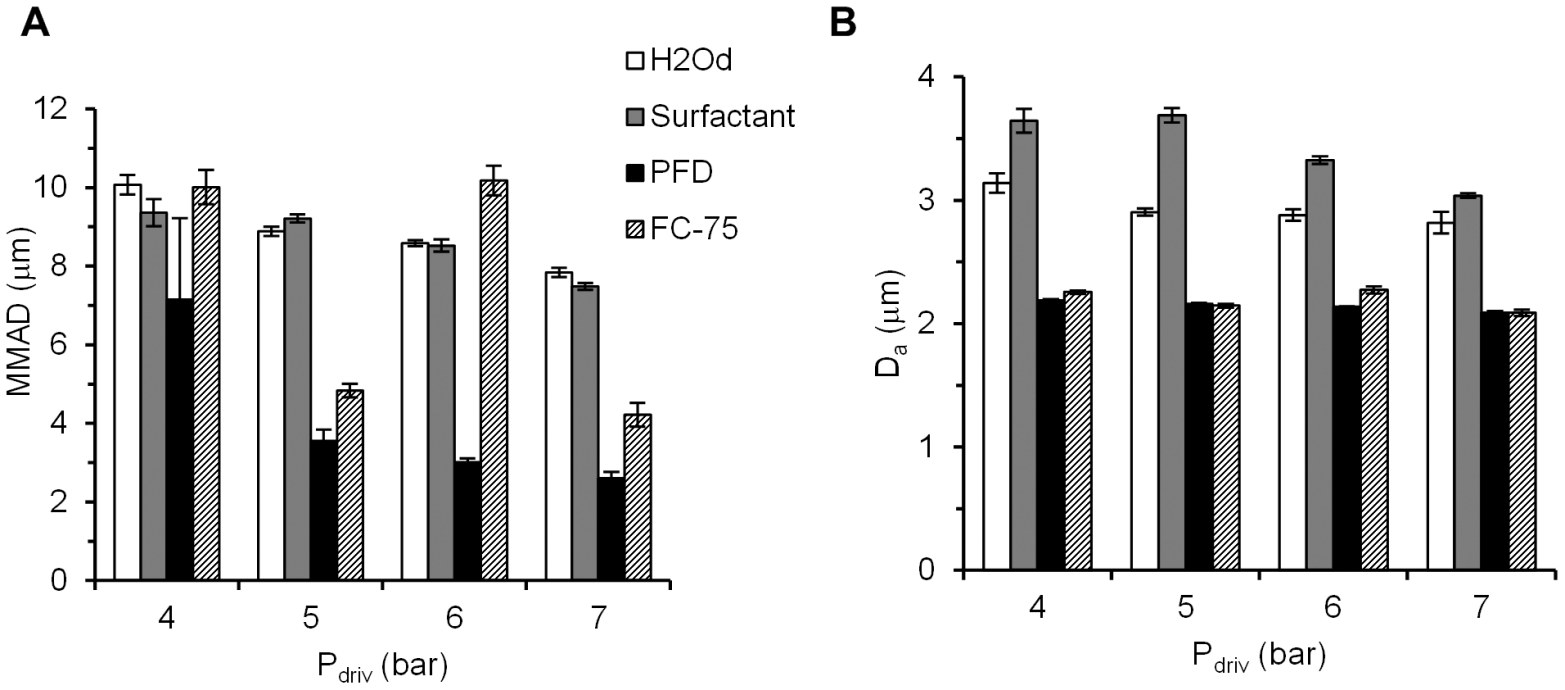
Particle size characterization parameters as a function of the aerosolized compound and the driving pressure. A, Mass median aerodynamic diameter, MMAD. B, mean aerodynamic diameter, D_a_. H_2_Od: distilled water. PFD: perfluorodecalin. P_driv_: driving pressure. Values are given as mean ± SD.

The IC produced heterodisperse aerosols with a GSD range of 1.93–2.51 for H_2_Od, 1.91–1.99 for surfactant, 1.50–1.57 for PFD and 1.56–1.69 for FC-75 (Table 1). The highest MMAD was registered after H_2_Od aerosolization at a driving pressure of 4 bar (10.07±0.25 µm), whereas the lowest value was achieved after PFD aerosolization at a driving pressure of 7 bar (2.61±0.15 µm) (Figure 10). Irrespective of the compound used, MMAD generally decreased if the driving pressure was increased. With FC-75, a MMAD of 10.18±0.38 µm was obtained at 6 bar, an extraordinarily high value compared to the values obtained at 5 and 7 bar. The reason for this could be the high volatility of PFCs.

**Table 1:** Geometric standard deviation (GSD) for different compounds and driving pressures.

	GSD			
P_driv_ (bar)	H_2_Od	Surfactant	PFD	FC-75
4	2.51±0.01	1.93±0.02	2.45±0.01	2.35±0.04
5	1.97±0.01	1.99±0.01	1.95±0.02	1.91±0.01
6	1.57±0.01	1.55±0.004	1.53±0.002	1.50±0.01
7	1.69±0.01	1.61±0.01	1.64±0.01	1.56±0.02

H_2_Od: distilled water. PFD: Perfluorodecalin. GSD: geometric standard deviation. P_driv_: driving pressure. Values are given as mean ± SD.

The D_a_ of all the aerosols was below 5 µm, regardless of the aerosolized compound or driving pressure. However, it was slightly higher in the case of H_2_Od and surfactant than in PFCs.

## Discussion

In the light of the results, this section highlights the main achievements of this work, whose aim was to demonstrate the feasibility of an intracorporeal aerosol-generating system to deliver large amounts of surfactant and PFC within and beyond third-generation branching. In addition, a comparison between the results presented here and other works is made and other interesting findings are presented. Finally, the main conclusion that supports the initial hypothesis as well as the limitations of the study are noted.

The deposition study indicates that it is feasible to generate relatively high amounts of therapeutic aerosols at the tip of a nasopharyngeal tube, and consequently this approach has the potential to significantly increase the residual pulmonary aerosol deposition in preterm and term neonates. This fact is reflected by the high percentage, namely between 65 and 85%, of the aerosolized mass that was measured at the exit of the printed model (E_+3_). Moreover, medical experts agree that if surfactant reaches the third generation, it would be successfully spread to the distal part of the lungs. The proposed intracorporeal aerosol delivery could be done in a relatively short time period. For instance, a standard porcine surfactant dose (200 mg/kg) for a 1,500 g infant could be delivered in just 10 minutes in contrast to the longer aerosolization times previously reported in the literature [7], [15], [28]. In the case of PFCs, recognizing that the ideal dose for aerosol delivery may vary depending on the PFC type [20], [23], [29], a dose of 10 ml/kg could be delivered to the same infant in just 10 minutes.

Another main finding was that produced aerosols had a mean aerodynamic diameter of between 2 and 4 µm, which is within the optimal range of 1–5 µm recommended by medical experts [30]. However, it is interesting to mention that the measured MMAD was between 2 and 10 µm, meaning that in some cases a high percentage of the aerosolized compound was transported by particles with a greater size than the optimal. Nevertheless, in our study the aerosol was generated at the beginning of the printed trachea model so the problem of depositing these great particles at the upper respiratory tract was avoided. On the other hand, a characteristic property of surfactant is its capacity to spread to lower generations due to its high viscosity, and so although these particles were deposited within the trachea, they would spread to the lower generations [31]. For these reasons, this particle size is acceptable in order to deliver surfactant to the lungs.

Finally, with regard to the high proximal pressure at which the catheter control system was operated, the pressure and the flow exerted by the IC were determined at its distal end in order to study the implications of the use of the IC during neonatal non-invasive respiratory support. Our measured air flow values produced by the IC are in line with those reported by the manufacturer, and they are smaller than the air flow values currently applied to neonates through a humidified nasal *cannula* [32]. Moreover, during a ventilator driven CPAP of 5 cmH_2_O, the increase in pressure attributable to the IC barely reached 1–2 cmH_2_O out of a maximum total CPAP of 6.73±0.04 cmH_2_O, a difference that may be corrected in the ventilator to maintain the pre-set CPAP level during aerosol delivery with the IC.

In order to compare our study with other works, an analysis related to the delivery method and the physical model we developed was done. Aerosol delivery of surfactant and PFC has been proposed as an alternative to their intratracheal instillation for the treatment of various pulmonary diseases, including neonatal RDS [9], [12], [20], [23], [33]. The main goal of aerosol therapy with surfactant and PFC for the treatment of this syndrome would be to administer these compounds during non-invasive ventilation with minimal airway manipulation. Most clinical studies with aerosolized surfactant, however, have failed to show a significant pulmonary effect of the aerosol therapy [7], [14], [15]; this may be partially justified by the level of lung deposition of therapeutic aerosols in preterm neonates, which has been reported to be very low, less than 1% of the nominal dose, irrespective of whether aerosol delivery was conducted during spontaneous breathing or during mechanical ventilation [17], [18]. In turn, this rather poor lung deposition may be partly explained by the low lung volumes of neonates, the caliber of their airways, and their fast and irregular respiratory rate [16], [34]. In addition, the performance of aerosol delivery devices is significantly affected by the use of surfactant and PFCs [19], [24], and moreover, extracorporeal aerosols must pass through the upper airways and even through the tubes of the respiratory support, which, given their narrow cross-section, leads to an increased non-pulmonary deposition of the aerosol. Experimental studies have shown an increased surfactant and PFC lung deposition and, therefore, a better pulmonary response if the aerosol therapy is conducted by intratracheal delivery, even improving the results achieved after instillation [20], [35]. These studies, however, were performed in animals intubated for mechanical ventilation, which, in turn, represents the main drawback currently associated with surfactant replacement therapy. One of the aims of the present study was precisely to test the feasibility of delivering intracorporeal surfactant and PFC aerosols in a *non-invasive* manner while maintaining the strict standards for neonatal care in terms of distal air flow and pressure, understanding non-invasive to refer to any kind of respiratory support that avoids ventilation through a tracheal tube, such as nasopharyngeal CPAP. For this reason, at the present *in vitro* set-up the placement of an IC into a nasopharyngeal tube has been simulated just above the vocal cords, which would make it possible to apply CPAP during aerosol delivery.

Airway models that take into account the anatomy and dimensions of infants have been of great value in aerosol deposition studies *in vitro*. Janssens et al. developed the SAINT upper airway model [36], using as starting point a computer tomography scan from the nasal bone to the subglottis (about 3 mm below the vocal cords) of a 9-month old infant. On the other hand, Minocchieri et al. developed the PrINT model [37] from a three-planar magnetic resonance imaging scan of the upper airways of a preterm infant of 32 weeks of gestational age. This model includes the upper airways from the nostrils down to 4 mm below the vocal cords. Although very valuable to test *in vitro* deposition of extra-corporeally generated aerosols, these models were of limited relevance for simulating the generation of an aerosol just above the vocal cords as was the objective in our study. Therefore, we developed a model of the trachea and three further generations in order to perform an *in vitro* aerosol deposition study of surfactant and PFC in the upper conducting airways of a neonate. We found a four generation model adequate for this study assuming that all surfactant and PFC deposited within or beyond the model will spread towards the lungs, as during intratracheal instillation.

Another interesting conclusion of the present study is that even though the studied method may allow non-invasive surfactant and PFC delivery during CPAP, the data from the calculation of the L_col_ of both surfactant and PFC aerosols predict the formation of a film of surfactant or PFC within the trachea during continuous aerosol delivery; hence, although non-invasive, it is debatable whether it could be considered a true aerosol therapy. The formation of the film was evident in the case of H_2_Od and surfactant; in these cases, the MMAD was the highest and the E_FP_ represented only a modest fraction of the total emission, whereas the highest amount of compound was collected beyond the model. In this scenario, it may be of concern that the delivery of a very high volume of medication to the conducting airways could lead to a transient obstruction and, in turn, to undesired peridosing episodes as during intratracheal instillation [38], [39]. This potential obstruction problem might be partially solved either by lowering the driving pressure of the catheter control system, which would reduce the surfactant and PFC aerosol production rate, or by performing the therapy in a non-continuous way. The first alternative, however, would increase the particle size and, therefore, might affect the distal lung deposition.

In the case of the PFCs, however, the IC achieved a far larger E_FP_ than with surfactant (up to 49.8±2.0%). Therefore, this delivery method is very interesting for PFC delivery since part of the PFC will be delivered as a film, achieving a good level of central lung deposition, and another part consisted of a large amount of smaller particles, as represented by the E_FP_ and by the lower MMAD values, may still reach the distal lung.

Comparing the two PFCs, the aerosol production rate (AR) for FC-75 was significantly higher than that observed for PFD. This fact might be explained by differences of the compounds in terms of vapor pressure, density, and especially, in kinematic viscosity [19], [24]. Specifically, the larger number of particles due to the higher aerosol production of FC-75, in comparison to PFD, might have accounted for the increased MMAD with this particular compound, likely due to particle coalescence. In order to optimize the PFC characteristics for aerosol delivery, in particular to achieve adequate aerosol production rates and particle size distributions, it may be necessary to test a wide range of PFC types with different physical-chemical properties [19], [33], and PFC combinations [40].

In conclusion, with this delivery system it is *a priori* feasible to deliver relatively large amounts of surfactant and PFC to the lungs in combination with non-invasive respiratory support under neonatal conditions by intracorporeal aerosol generation, as was initially hypothesized. Nevertheless, this study has a number of limitations that must be acknowledged. On the one hand, the *in vitro* set-up represents the ideal conditions for intracorporeal aerosol delivery with the IC in the same vertical axis of the trachea; *in vivo*, it might be difficult to achieve an ideal placement of the IC and therefore, a significant fraction of the aerosol would deposit in the nasopharyngeal region or be redirected to the oesophagus. Further, the model did not account for the temperature and humidity of the respiratory system and we used a constant inspiratory flow, without exhalation. With these limitations in mind, an increased deposition of surfactant and PFC within the lungs can, nevertheless, be expected if an aerosol is generated intracorporeally by means of an aerosol delivery system properly customized for neonates.

## Supporting Information

File S1 **Abstract of a previous work used to build the initial hypothesis.**
(PDF)

File S2 **Summary of the results obtained from the analysis of the data.**
(XLSX)

File S3 **Raw data obtained from particle size distribution measurements.**
(RAR)

File S4 **Raw data obtained from pneumotach measurements.**
(RAR)
