# Supplementary material for: In Vitro Surfactant and Perfluorocarbon Aerosol Deposition in a Neonatal Physical Model of the Upper Conducting Airways
Source: PLoS One. 2014 Sep 11;9(9):e106835. doi: 10.1371/journal.pone.0106835 (PMC4161382; doi:10.1371/journal.pone.0106835)
Supplement: File S1 — Abstract of a previous work used to build the initial hypothesis. (PDF) [file pone.0106835.s001.pdf]

549

ACUTE AND SUSTAINED EFFECTS OF AEROSOLIZED VS. BOLUS ADMINISTRATION SURFACTANT ON PULMONARY MECHANICS AND SYSTEMIC AND CEREBRAL HAEMODINAMIC IN PREMATURE LAMBS WITH RESPIRATORY DISTRESS SYNDROME

C. Rey-Santano[1], V.E. Mielgo[1], E. Gastiasoro[1], F.J. Alvarez[1], H. Lafuente[1], E. Ruiz-del-Yerro[1], S. Gomez[2], L. Andres[3], A. Valls-i-Soler[4], X. Murgia[1]

[1]Research Unit, Cruces Hospital, Barakaldo, [2]Neurosciences, Basque Country University, Leioa, [3]Department of Pathology, [4]Neonatal Unit, Cruces Hospital, Barakaldo, Spain

Background: In neonatal RDS bolus administration of surfactant (SF) is an effective therapy, however has been linked to “peridosing adverse events”. Aerosolization of SF has emerged as feasible alternative to instillation for RDS.

Aim: To determinate the effects of aerosolized SF on gas exchange, lung function and cerebral hemodynamic in preterm lambs.

Methods: 21 preterm lambs (85%gestation) were randomly assigned to receive aerosolized SF (Curosurf ®, 200mg/kg in 20min), delivered via an inhalation catheter coupled to a jet nebulizer and connected to a ventilator (SF-Aero), bolus SF (SF-Bolus) or not (Control), groups were maintained during 6h in IMV. Gas exchange [oxygenation index (OI) and P_{aCO2}], systemic and cerebral hemodynamic [mean arterial blood pressure (MABP), heart rate (HR) and cerebral blood flow (CBF)], lung mechanics [compliance (C_{dyn}) and tidal volume (V_T)] were measured each 30 min. Mean±SD, ANOVA, $p< 0.05$.

Results: After 60min of treatment, animals in SF-Aero and SF-Bolus groups, significantly improved OI, P_{aCO2}, C_{dyn} and V_T in comparison to control group, being improvement persistent until the end of the experiment. Also, there was a significant increase of P_{aCO2} and CBF in SF-Bolus group ($p< 0.05$) during the first 30 min of treatment, without changes on MABP and HR in comparison with SF-Aero group, in which there was a gradual decrease of P_{aCO2} and CBF along the time.

Conclusion: In preterm lambs with RDS, the SF aerosolization improves pulmonary function and gas exchange without evidenced of acute of sustained hemodynamic changes thus; this SF administration technique is effective and safe.
